# *In vivo* microsampling to capture the elusive exposome

**DOI:** 10.1038/srep44038

**Published:** 2017-03-07

**Authors:** Vincent Bessonneau, Jennifer Ings, Mark McMaster, Richard Smith, Leslie Bragg, Mark Servos, Janusz Pawliszyn

**Affiliations:** 1 Department of Chemistry, University of Waterloo, ON, Canada; 2 Water Science and Technology Directorate, Environment Canada, Burlington, ON, Canada; 3 Mass Spectrometry Facility, University of Waterloo, ON, Canada; 4 Department of Biology, University of Waterloo, ON, Canada

## Abstract

Loss and/or degradation of small molecules during sampling, sample transportation and storage can adversely impact biological interpretation of metabolomics data. In this study, we performed *in vivo* sampling using solid-phase microextraction (SPME) in combination with non-targeted liquid chromatography and high-resolution tandem mass spectrometry (LC-MS/MS) to capture the fish tissue exposome using molecular networking analysis, and the results were contrasted with molecular differences obtained with *ex vivo* SPME sampling. Based on 494 MS/MS spectra comparisons, we demonstrated that *in vivo* SPME sampling provided better extraction and stabilization of highly reactive molecules, such as 1-oleoyl-sn-glycero-3-phosphocholine and 1-palmitoleoyl-glycero-3-phosphocholine, from fish tissue samples. This sampling approach, that minimizes sample handling and preparation, offers the opportunity to perform longitudinal monitoring of the exposome in biological systems and improve the reliability of exposure-measurement in exposome-wide association studies.

Since genetic factors typically account for only about 18% of chronic disease risks^1^ exposome-wide association studies (EWAS) that measure entire classes of small molecules in biospecimens (resulting from myriad of environmental exposures) are being conducted to discover unknown causes of chronic diseases^2^. In biological systems, small molecules can be either substrates or end products of cellular metabolism and can originate from exogenous sources via a myriad of exposures, or from endogenous processes including host and microbial metabolism. Most EWAS rely on untargeted metabolomics analysis of biospecimens from incident disease cases and matched controls to measure hundreds or thousands of features that then generate candidate discriminating biomarkers^3^. The choice of sampling, sample collection, and sample preparation strategy plays an important role in the quality of metabolomics data^4^. Issues of incomplete metabolism quenching, ionization suppression, and metabolite instability have been well-documented and can adversely impact data interpretation. One way to circumvent these issues is to use *in vivo* sample preparation techniques that provides a true(r) representation of the exposome by eliminating some of the variability introduced in sample depletion and multistep sample handling^5^,^6^,^7^,^8^.

An example of such sample preparation technique is the sorbent-coated device, solid-phase microextraction (SPME). Over the past years, *in vivo* SPME has been successfully applied for targeted and untargeted metabolomics analysis of various biospecimens^9^ owing to the biocompatibility of SPME coatings. *In vivo* SPME combine sampling, metabolite extraction, and metabolism quenching in one step, limiting loss and/or degradation of metabolites^9^. We have recently demonstrated that it is possible to extract hundreds of chemicals with a single coated fiber from plasma, including short-lived and unstable metabolites not detected and/or quantified accurately with other techniques^10^,^11^.

Here we performed *in vivo* SPME in combination with liquid chromatography (LC) and data-dependent tandem high-resolution mass spectrometry (MS/MS) to capture the fish tissue exposome, and examined molecular differences obtained with *ex vivo* SPME analysis from the same biospecimens using molecular networking analysis. *In vivo* extraction of metabolites from fish tissue (n = 60, white sucker) was achieved by inserting a PAN-C18 coated SPME blade into the dorsal-epaxial muscle for 20 min. *Ex vivo* SPME sampling was conducted using the same procedure but from tissue samples collected after fish euthanasia and being stored frozen. Extracted molecules were desorbed in an 80% v/v acetonitrile solution, and analyzed with reversed-phase LC-MS/MS using a pentafluorophenyl column and a Q-Exactive Quadrupole-Orbitrap MS in positive ionization mode (Detailed description of experimental conditions can be found in Supplementary Information).

## Materials and Methods

### Study design

**Figure 1:**
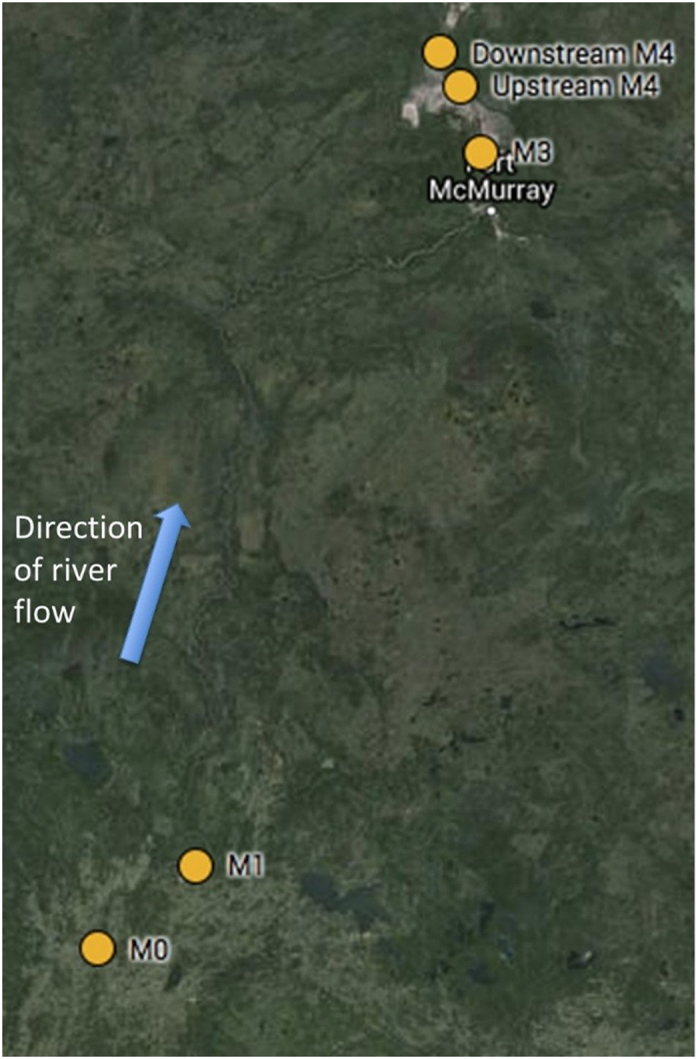
Map of different sites investigated generated from Google. (n.d.). [Google Maps of fish collection sites in Alberta, Canada]. Map data ©2016 Google. Retrieved September 17, 2016, from https://goo.gl/HBAeBS.

Adult white sucker (*Catostomus commersonii*) (40.8 ± 3.6 cm, 969.7 ± 303.3 g, *n* = 60) were collected by boat electrofishing from the Athabasca River in the Alberta oil sands region (Northern Alberta, Canada). As part of a larger sampling effort, twelve fish were collected in September 2013 from: 2 sites outside of the deposit, M0 (Athabasca) and M1, which are both downstream of a pulp and paper mill discharge; 1 site upstream of the oil sands development but within the deposit around Northlands Sawmill (Downstream of M3); 1 site adjacent to the oil sands development (Upstream of M4); and 1 site downstream of the Muskeg River within the deposit and downstream of the development (Downstream M4) (Fig. 1). In total, a subset of 6 males and 6 females were selected at each site and held briefly (<1 h) in cages in the river until sampling.

### SPME blade coating preparation

PAN-C18 SPME blades were prepared as previously described^12^ by immobilization of particles on the surface of stainless steel blades. Briefly, 5 μm particles (Supelco, PA) were immobilized (60 μm coating thickness) using a polyacrylonitrile (PAN) solution which acted as a bonding agent.

### *In vivo* and *ex vivo* SPME sampling procedure of fish tissue

All experimental protocols were in accordance with and as approved by the University of Waterloo Animal Care Committee (AUPP #10–17). *In vivo* extraction of metabolites from fish tissue was conducted by inserting a PAN-C18 SPME coated blade into the dorsal-epaxial muscle (near the dorsal fin) of fish immobilized using a large foam bed^13^,^14^. The blade remained in place for 20 min while fish were held in an aerated, 28 L covered bucket. After 20 min, the blade was removed, rinsed with nanopure water to remove matrix components, and frozen in liquid nitrogen. Fish were then sacrificed and a small part of the dorsal-epaxial muscle was cut out, placed in aluminum foil and frozen in liquid nitrogen on-site and shipped to our laboratory. In the laboratory, *ex-vivo* SPME sampling was performed by inserting a PAN-C18 coated blade into a thawed, non-homogenized tissue sample for 20 min without agitation. Desorption of metabolites from the SPME coating of the blades was done by immersing them for 60 min in 1 mL of acetonitrile/water (80/20, v/v) with vortex agitation at 1000 rpm. Extracts were stored at −80 °C until analysis.

### UPLC-Q-Exactive Orbitrap HRMS analysis

Metabolite profiling was conducted using an LC-MS system consisting of a ThermoAccela autosampler, pumps and a Q-Exactive Orbitrap System (Thermo Fisher Scientific, CA, USA). Metabolites were separated by a reversed-phase method using a pentafluorophenyl column (Kinetex Phenomenex, 2.1 mm × 100 mm, 1.7 μm particle size) at a flow rate of 300 μL/min. Mobile phase A consisted of water/formic acid (99.9/0.1, v/v) and mobile phase B consisted of acetonitrile/formic acid (99.9/0.1, v/v). The starting mobile phase conditions were 90% A from 0 to 1.0 min, followed by a linear gradient to 10% A from 1.0 to 9.0 min and an isocratic hold at 10% A until 12.0 min. The total run time was 18 min per sample, including a 6 min re-equilibration time. The injection volume was 10 μL. Analyses were performed in positive ionization mode in the mass range of m/z 50–750. To maintain a mass accuracy better than 5 ppm, we used the following lock mass: m/z 391.2843. Instrument parameters were set as follows: sheath gas (Nitrogen) flow rate, 35 arbitrary units; capillary voltage, 3.1 kV; ion source temperature 280 °C; full MS automatic gain control (AGC), 1.10^6^; spectra rate acquisition, 3.7 spectra/s; full MS resolution, 70,000. MS/MS fragmentation of the ten most intense ions per spectrum was performed using a normalized collision energy (NCE) of 50; MS/MS resolution, 17,500; MS/MS AGC, 2.10^5^; precursor ion mass isolation window, 1 ppm. MS/MS exclusion list was set after 3 analyses of blank SPME samples.

### UPLC-MS/MS data processing and molecular networking

Molecular networking of LC-MS/MS data was performed using the Global Natural Products Social Molecular Networking (GNPS) software^15^. For MS and MS/MS spectral library search and molecular networking, we used an ion mass tolerance of 1 and 0.01 Da for precursor ions and fragment ions, respectively. A minimum cosine similarity score of 0.7 was used for MS/MS spectral library matching. A minimum cosine similarity score of 0.7 and a minimum number of 6 matched fragment ions were used to form a network of two consensus MS/MS spectra. Resulting molecular networks were built and visualized using Cytoscape 3.2.1^16^. Precursor ion m/z was used as node attribute and cosine similarity score was used as edge attribute.

## Results and Discussion

**Figure 2:**
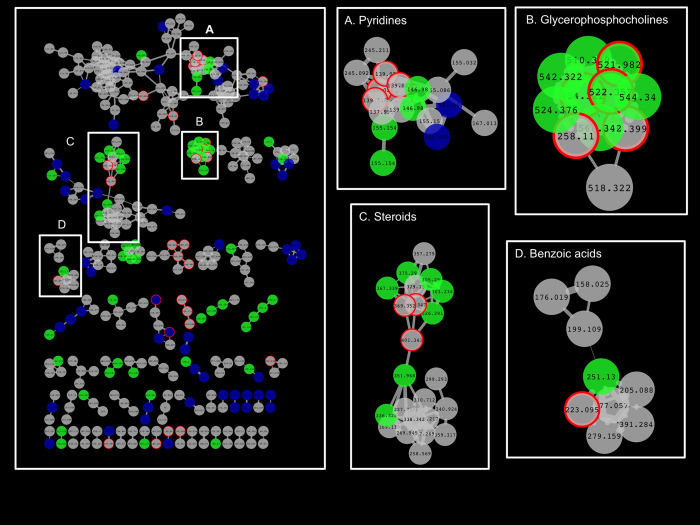
Chemical-similarity maps of small molecules (n = 494 with MS/MS spectrum) in fish tissue (Tanimoto coefficient ≥0.7). Green and blue nodes represent small molecules observed only after *in vivo* SPME sampling and *ex vivo* SPME sampling, respectively. Grey nodes represent small molecules detected using both sampling methods. Nodes with red border indicates annotated molecule by matching LC-MS/MS libraries with spectral similarity ≥0.7 Edge represents similarity between MS/MS spectra. Thickness of the edges indicates the level of similarity (the thicker is an edge, the more similar are MS/MS spectra).

After molecular networking of fish tissue LC-MS/MS data, we found 494 nodes representing consensus of at least two or more MS/MS spectra (Fig. 2). The majority of the nodes matched chemicals detected in fish tissue using both sampling approaches. Only 7% of them could be identified by matching LC-MS/MS libraries with spectral similarity ≥0.7 (Table 1), suggesting that the vast majority of chemicals in fish tissue are unknown. However, mapping the chemical similarity between unknown and identified molecules can help uncover chemical classes of the uncharacterized molecules. Although the majority of identified chemicals were endogenous molecules, four molecules including 4-methoxycinnamic acid, 1-hydroxybenzotriazole, diethylphthalate, and phenoxybenzamine used in the formulation of sunscreen products^17^ dishwasher cleaning products^18^ and plasticizers^19^ or as active ingredients in pharmaceutical products^20^ respectively, were detected in fish tissues. These chemicals probably originated from external exposures (i.e. water contamination), confirming that fish tissue offers opportunity to reconstruct past environmental exposures due to bioaccumulation of persistent organic toxicants, and can be used to monitor aquatic ecosystem health.

We then evaluated the ability of *in vivo* SPME to capture unstable molecules. We observed that 16% of the nodes were only detected after *in vivo* SPME sampling, while 21% only found after *ex vivo* SPME sampling. Molecules only observed with *in vivo* SPME include cinnamic acids, glycerophosphocholines, phenylmethylamines, phenoxybenzamines and pyridincarboxylic acids originating from both exogenous and endogenous exposures (Table 1). For example, 1-oleoyl-sn-glycero-3-phosphocholine is a lipid-signaling molecule generated by phospholipase enzymes^21^. This compound contains an unsaturated acyl chain where the hydrogen atom on methylene groups adjacent to the double bounds has low carbon-hydrogen (C-H) bond energies, and is therefore a major target for modification under oxidative conditions after sample collection^22^. Five other glycerophosphocholines (Fig. 1B; m/z 542.322, m/z 524.376, m/z 568.342, m/z 544.340, and m/z 510.359) were only detected using *in vivo* SPME, but was not successfully identified due to the lack of adequate similarity (cosine ≥0.7) between their MS/MS spectra and those from public LC-MS/MS libraries. This result possibly indicates that these molecules have not been previously identified due to their chemical instability or experimental MS/MS spectra produced were a composite of two or more molecules. Similarly, cinnamic acids, phenylmethylamines, phenoxybenzamines and pyridincarboxylic acids only detected after *in-vivo* SPME are also highly reactive compounds prone to auto-oxidation during sample transportation and storage. However, glycerophosphocholines containing saturated acyl chains, such as 1-palmitoyl-sn-glycero-3-phosphocholine, which was detected after both *in vivo* and *ex vivo* SPME, are more resistant to oxidation^22^. Some molecules were only observed after *ex-vivo* SPME, including 6-hydroxynicotinate, hydroxyproline and 2-heptyl-3-hydroxy 4-quinolone. Previous studies have demonstrated that 6-hydroxynicotinate and 2-heptyl-3-hydroxy 4-quinolone are essentially produced from bacterial metabolism^23^,^24^ and possibly originated from tissue degradation during sample transportation and storage. Hydroxyproline, a major constituent of collagen, is also used as an indicator of tissue damage or degradation^25^.

## Conclusion

In summary, we demonstrated that *in vivo* SPME sampling provided extraction and stabilization of highly reactive chemicals, not detected by *ex vivo* sample preparation techniques due to their auto-oxidation during sample collection, transportation and storage. *In vivo* SPME sampling provides extraction of molecules with a wide range of chemical and physical properties^9^ (balanced coverage), which originate from exogenous sources via environmental exposures or from endogenous processes including host and microbial metabolism. *In vivo* SPME sampling as a minimally invasive technique also offers the opportunity to perform repeated sampling over time on the same subject, limiting inter and intra-individual variability in levels of circulating molecules arising from changes in environmental factors (e.g. diet or sources of pollutants).

## Additional Information

**How to cite this article:** Bessonneau, V. *et al*. *In vivo* microsampling to capture the elusive exposome. *Sci. Rep.* **7**, 44038; doi: 10.1038/srep44038 (2017).

**Publisher's note:** Springer Nature remains neutral with regard to jurisdictional claims in published maps and institutional affiliations.

## Figures and Tables

**Table 1:** Small molecules identified in fish tissue using solid-phase microextraction combined with LC-MS/MS with a Q-Exactive Quadrupole-Orbitrap mass spectrometer.

Metabolite	Family	Precursor m/z	Precursor adduct	RT (min)	Sample class	Similarity cosine^a^
4-methoxycinnamic acid	Cinnamic acids	179.070	[M + H]+	8.48	G2	0.78
Phenoxybenzamine	Phenylmethylamines	304.147	[M + H]+	6.98	G2	0.82
Nicotinamide	Pyridinecarboxylic acids	123.055	[M + H]+	0.86	G2	0.83
1-Oleoyl-sn-glycero-3-phosphocholine	Glycerophosphocholines	522.355	[M + H]+	7.5	G2	0.87
1-Palmitoleoyl-glycero-3-phosphocholine	Glycerophosphocholines	494.324	[M + H]+	6.78	G2	0.70
4-Chlorophenol	Chlorophenols	129.010	[M + H]+	0.70	G1	0.74
Hydroxyproline	Carboxylic acids and derivates	132.065	[M + H]+	0.88	G1	0.83
6-Hydroxynicotinate	Pyridinecarboxylic acids	140.034	[M + H]+	0.97	G1	0.78
2-Heptyl-3-hydroxy 4-quinolone	Quinolones	260.165	[M + H]+	8.30	G1	0.78
Phenylalanine	Phenylpropanoic acids	166.087	[M + H]+	1.38	G1, G2	0.96
Tyrosine	Phenylpropanoic acids	182.081	[M + H]+	0.85	G1, G2	0.97
Deoxycarnitine	Fatty acids and conjugates	146.118	[M + H]+	0.99	G1, G2	0.99
1-Hydroxybenzotriazole	Benzotriazoles	136.049	[M + H]+	0.90	G1, G2	0.70
Creatine	Carboxylic acids and derivates	132.077	[M + H]+	0.88	G1, G2	0.97
3-Methylhistidine	Carboxylic acids and derivates	170.093	[M + H]+	0.92	G1, G2	0.82
Cholesterol	Steroids	369.352	[M−H_2_O + H]+	10.1	G1, G2	0.95
Histidine	Carboxylic acids and derivates	156.078	[M + H]+	0.90	G1, G2	0.99
Tryptophan	Indoles	205.097	[M + H]+	2.90	G1, G2	0.93
Arginine	Carboxylic acids and derivates	175.119	[M + H]+	0.94	G1, G2	0.95
2-amino-2-methylpropanoate	Carboxylic acids and derivates	106.050	[M+H]+	0.74	G1, G2	0.98
Proline	Carboxylic acids and derivates	116.071	[M+H]+	0.76	G1, G2	0.87
Betaine	Carboxylic acids and derivates	118.086	[M+H]+	0.75	G1, G2	0.95
Threonine	Carboxylic acids and derivates	120.066	[M+H]+	0.74	G1, G2	0.91
L-Glutamine	Carboxylic acids and derivates	147.076	[M+H]+	1.01	G1, G2	0.95
L-Methionine	Carboxylic acids and derivates	150.058	[M+H]+	0.84	G1, G2	0.81
Carnitine	Fatty acids and conjugates	162.112	[M+H]+	0.95	G1, G2	0.94
O-Acetylcarnitine	Fatty acids and conjugates	204.123	[M+H]+	1.25	G1, G2	0.96
Diethylphthalate	Benzoic acids	223.096	[M+H]+	6.22	G1, G2	0.90
Inosine	Purine nucleosides	269.088	[M+H]+	0.80	G1, G2	0.94
Adenosine	Purine nucleosides	268.103	[M+H]+	0.89	G1, G2	0.97
Guanosine	Purine nucleosides	284.099	[M+H]+	0.80	G1, G2	0.91
Inosinic acid	Purine nucleotides	349.054	[M+H]+	0.72	G1, G2	0.88
Desmosterol	Steroids	385.346	[M+H]+	7.98	G1, G2	0.75
3β-Hydroxy-5-cholestenal	Steroids	401.341	[M+H]+	8.88	G1, G2	0.80
sn-Glycero-3-phosphocholine	Glycerophosphocholines	258.110	[M+H]+	0.70	G1, G2	0.94
1-Palmitoyl-sn-glycero-3-phosphocholine	Glycerophosphocholines	496.339	[M+H]+	7.3	G1, G2	0.89
1-Palmitoyl-sn-glycero-3-phosphocholine	Glycerophosphocholines	518.322	[M+Na]+	7.3	G1, G2	0.89

^a^Cosine similarity value between experimental MS/MS spectrum and MS/MS spectrum from public LC-MS/MS libraries.

G1: *ex-vivo* SPME sampling; G2: *in-vivo* SPME sampling.

## References

[b1] Rappaport S. M. Genetic factors are not the major causes of chronic diseases. PLoS ONE 11, e0154387 (2016). 27105432 10.1371/journal.pone.0154387 PMC4841510

[b2] Wild C. P. Complementing the genome with an “exposome”: The outstanding challenge of environmental exposure measurement in molecular epidemiology. Cancer Epidemiol. Biomarkers Prev. 14, 1847–1850 (2005). 16103423 10.1158/1055-9965.EPI-05-0456

[b3] Rappaport S. M. Implications of the exposome for exposure science. J. Expo. Sci. Environ. Epidemiol. 21, 5–9 (2011). 21081972 10.1038/jes.2010.50

[b4] Ryan D. & Robards K. Metabolomics: The greatest omics of them all? Anal. Chem. 78, 7954–7958 (2006). 17134127 10.1021/ac0614341

[b5] Bruce S. J., Tavazzi I., Parisod V., Kochlar S. & Guy P. A. Investigation of human blood plasma sample preparation for performing metabolomics using ultrahigh performance liquid chromatography/mass spectrometry. Anal. Chem. 81, 3285–3296 (2009). 19323527 10.1021/ac8024569

[b6] Moco S., Vervoort J., Bino R. J., De Vos R. C. H. & Bino R. Metabolomics technologies and metabolite identification. Trends Anal. Chem. 26, 855–866 (2007).

[b7] Theodoridis G., Gika H. G. & Wilson I. D. LC-MS-based methodology for global metabolite profiling in metabonomics/metabolomics. Trends Anal. Chem. 27, 251–260 (2008).

[b8] Canelas A. B. . Quantitative evaluation of intracellular metabolite extraction techniques for yeast metabolomics. Anal. Chem. 81, 7379–7389 (2009). 19653633 10.1021/ac900999t

[b9] Bojko B. . Solid-phase microextraction in metabolomics. Trends Anal. Chem. 61, 168–180 (2014).

[b10] Vuckovic D. . *In vivo* solid-phase microextraction: Capturing the elusive portion of metabolome. Angew. Chem. Int. Ed. 50, 5344–5348 (2011). 10.1002/anie.201006715 21509917

[b11] Bessonneau V., Zhan Y., De Lannoy I. A. M., Saldivia V. & Pawliszyn J. *In vivo* solid-phase microextraction liquid chromatography-tandem mass spectrometry for monitoring blood eicosanoids time profile after lipopolysaccharide-induced inflammation in Sprague-Dawley rats. J. Chromatogr. A 1424, 134–138 (2015). 26585209 10.1016/j.chroma.2015.10.067

[b12] Mirnaghi F. S., Chen Y., Sidisky L. M. & Pawliszyn J. Optimization of the coating procedure for a high-throughput 96-blade solid-phase microextraction system coupled with LC-MS/MS for analysis of complex samples. Anal. Chem. 83, 6018–6025 (2011). 21711040 10.1021/ac2010185

[b13] Ouyang G. . Sampling-rate calibration for rapid and nonlethal monitoring of organic contaminants in fish muscle by solid-phase microextraction. Environ. Sci. Technol. 45, 7792–7798 (2011). 21838320 10.1021/es201709j

[b14] Zhang X., Oakes K. D., Wang S., Cui S. & Pawliszyn J. *In vivo* sampling of environmental organic contaminants in fish by solid-phase microextraction. Trend Anal. Chem. 32, 31–39 (2012).

[b15] Wang M. . Sharing and community curation of mass spectrometry data with Global Natural Products Social Molecular Networking. Nature Biotechnology 34, 828–837 (2016). 10.1038/nbt.3597 PMC5321674 27504778

[b16] Shannon P. . Cytoscape: A software environment for integrated models of biomolecular interaction networks. Genome Res 13, 2498–2504 (2003). 14597658 10.1101/gr.1239303 PMC403769

[b17] Smith G. J. & Miller I. J. The effect of molecular environment on the photochemistry of *p*-methoxycinnamic acid and its esters. J. Photochem. Photobiol. A 118, 93–97 (1998).

[b18] Janna H., Scrimshaw M. D., Williams R. J., Churchley J. & Sumpter J. P. From dishwasher to tap? Xenobiotic substances benzotriazole and tolyltriazole in the environment. Environ. Sci. Technol. 45, 3858–3864 (2011). 21524137 10.1021/es103267g

[b19] Sánchez-Avila J., Tauler R. & Lacorte S. Organic micropollutants in coastal waters from NW Mediterranean Sea: sources distribution and potential risk. Environ. Int. 46, 50–62 (2012). 22706016 10.1016/j.envint.2012.04.013

[b20] Guzzetta N. A. Phenoxybenzamine in the treatment of hypoplastic left heart syndrome: A core review. Anesth. Analg. 105, 312–315 (2007). 17646482 10.1213/01.ane.0000275185.44796.92

[b21] Mustranta A., Forsell P., Aura A. M., Suortti T. & Poutanen K. Modification of phospholipids with lipases and phospholipases. Biocatalysis 9, 181–194 (1994).

[b22] Reis A. & Spickett C. M. Chemistry of phospholipid oxidation. Biochim. Biophys. Acta 1818, 2374–2387 (2012). 22342938 10.1016/j.bbamem.2012.02.002

[b23] Hurh B., Ohshima M., Yamane T. & Nagasawa T. Microbial production of 6-hydroxynicotinic acid, an important building block for the synthesis of modern insecticides. J. Ferment. Bioeng. 77, 382–385 (1994).

[b24] Diggle S. P. . The Pseudomonas aeruginosa 4-quinolone signal molecules HHQ and PQS play multifunctional roles in quorum sensing and iron entrapment. Chem. Biol. 14, 87–96 (2007). 17254955 10.1016/j.chembiol.2006.11.014

[b25] Nogueira Ade C., Vale R. G., Gomes A. L. & Dantas E. H. The effect of muscle actions on the level of connective tissue damage. Res. Sports Med. 19, 259–270 (2011). 21988268 10.1080/15438627.2011.608046

